# The low data fidelity of human-based informatics in surgical instrument waste reduction: is it time for objective computer vision integration?

**DOI:** 10.1186/s12913-026-14290-y

**Published:** 2026-03-06

**Authors:** Abner Mácola, Katie Poole, Jinan Sous, Andrew R. Bradley, Peter F. Nichol

**Affiliations:** 1 Department of Surgery, University of Wisconsin School of Medicine, and Public Health, H4/746 CSC 600 Highland Ave., Madison, WI 53792 USA; 2 Henry Predolin College of Nursing, Edgewood University, 1000 Edgewood College Drive, Madison, WI 53711 USA

**Keywords:** Human-based informatics, Surgical instruments, Waste reduction, Data fidelity

## Abstract

**Background:**

Unused but sterilized surgical instruments generate substantial financial and environmental waste. Tray optimization commonly relies on human-based education, feedback, and manual documentation, yet data fidelity remains uncertain.

**Methods:**

We conducted a retrospective pre–post observational analysis of a human-based tray optimization initiative involving six surgeons across multiple procedures. Utilization was defined as the ratio of instruments used to those opened per case. Adherence, data capture, and variability across surgeons and procedures were assessed.

**Results:**

Mean utilization increased from 0.335 to 0.548 (+ 63.6%). However, changes varied widely across surgeons (+ 0.103 to + 0.418), and one procedure showed decreased utilization. Critically, data capture was low and declined from 24.7% to 15.3%, indicating inconsistent participation and limited data fidelity.

**Conclusions:**

Despite improved average utilization, poor data fidelity, participation imbalance, and outcome variability constrain interpretation of human-based optimization efforts. Objective, automated approaches such as computer vision may support more reliable and scalable waste reduction.

## Introduction

The practice of opening sterilized surgical instrument trays containing items that are ultimately unused represents a major source of avoidable cost and environmental impact in healthcare [1, 2]. Unused instruments contribute to repeated sterilization cycles, increased labor demands, and downstream environmental impacts associated with energy, water, and material consumption across the perioperative supply chain [3]. As a result, instrument tray optimization has emerged as a common strategy aimed at aligning tray contents more closely with the actual needs of specific procedures and surgeons [4].

Most tray optimization initiatives reported in the literature rely on human-centered approaches, including staff education, surgeon feedback, and manual documentation of instrument use [5]. These interventions are intended to influence behavior and generate data to guide tray redesign. However, operating rooms are complex, high-acuity environments in which human observation, compliance, and reporting may be inconsistent [6]. Prior studies have noted that manual data collection in such settings is vulnerable to incomplete capture, reporting errors, and variable adherence, all of which may affect the reliability and interpretability of optimization efforts.

Despite widespread adoption of human-based tray reduction strategies, limited attention has been given to the reliability of the underlying data used to evaluate their effectiveness. In particular, few studies have examined the extent to which incomplete participation, inconsistent reporting, and variability across surgeons and procedures may influence observed outcomes. As a result, it remains unclear whether reported improvements in utilization reflect durable system-level change, improved data accuracy, or selective participation by highly engaged individuals [2–8].

This study examines a human-based tray optimization initiative using a pre- and post-intervention observational design, with particular emphasis on utilization outcomes, data fidelity, and adherence across surgeons and procedures. Rather than focusing solely on average changes in instrument use, the analysis evaluates variability and completeness of data capture to assess the reliability of human-centered approaches for sustainable waste reduction. The findings are used to inform whether more objective, automated data collection strategies may be necessary to support scalable and reproducible instrument optimization in the operating room (OR) [9, 10].

## Methods

### Study design and participants

This study was a retrospective, pre- and post-intervention observational analysis conducted at a single academic medical center. The intervention consisted of a human-based tray optimization initiative, including education, feedback, and manual review of instrument utilization, aimed at reducing the number of unused instruments opened per surgical case.

Data were collected from six surgeons across four commonly performed procedures: Appendectomy (Appe), Gastrostomy Tube placement (GT), Inguinal Hernia Repair (IHR), and Umbilical Hernia Repair (UHR). These procedures were selected based on their high procedural volume and the relatively low variability in instrument requirements between cases, making them appropriate candidates for standardized tray optimization efforts.

Only sterilized surgical instrument sets (trays) were included in the analysis. Individually wrapped instruments and disposable items opened outside of standardized trays were excluded, as the optimization initiative specifically targeted reusable instrument sets managed by sterile processing.

The study period was divided into pre-intervention and post-intervention phases to assess changes in utilization following implementation of the human-based optimization strategy.

### Data collection and processing

Instrument utilization data were recorded manually by nursing and surgical technician staff at the conclusion of each procedure using standardized count sheets for the relevant instrument sets. For each tray, the total number of instruments available and the number used during the procedure were documented. These records were subsequently transferred into a digital spreadsheet (Microsoft Excel) for analysis.

The primary outcome metric, utilization, was calculated at the case level as:

Utilization = Total instruments used / Total instruments available.

Higher utilization values indicated a greater proportion of opened instruments being used, reflecting reduced waste.

Case-level utilization values were aggregated by surgeon and by procedure type for both the pre- and post-intervention phases.

### Analysis of data fidelity, adherence, and utilization change

Adherence was assessed by comparing the number of procedures contributed by each surgeon in the Pre and Post phases. The primary outcome measure was the Delta in utilization, calculated as UtilizationPost - UtilizationPre. A positive Delta indicates a desirable increase in utilization (less waste), while a negative Delta indicates a decrease (more waste).

Subsequent analysis focused on examining the reliability of the human-based approach through:

1. Poor Data Fidelity: Highlighting the low and declining data capture rates (percentage of total numbers of a case types for which data was captured during the Pre and Post Phases) as a primary indicator of the study’s reliability or lack thereof.
2. Inconsistent Adherence and Data Imbalance: Quantifying the imbalance in case volumes and the concentration of data from a few surgeons to show that the dataset is not representative.
3. Variability in Outcome: Analyzing the range of Delta across different surgeons and procedures to determine if there was a consistent, predictable effect from the human-based intervention.

## Results

### Critically low data fidelity and inconsistent participation

The most compelling finding in this human-based study was the initially low and declining data capture rate. In the pre-intervention phase, only 24.7% of all possible cases were captured. This rate deteriorated further in the post-intervention phase, falling to just 15.3%. Such low fidelity undermines the validity of any conclusions drawn from the data.

Furthermore, the collected data was not balanced. A majority of the cases came from just two surgeons, who together accounted for 19 of the 36 total captured cases (53%). This imbalance, coupled with the significant variation in case volume across all participants (Table 1), demonstrates inconsistent adherence to the study protocol. The imbalance in case counts (e.g., Surgeon 1 with 5 Pre vs. 1 Post) compromises the validity of the paired comparison and highlights the difficulty of maintaining consistent human participation in such studies.

**Table 1:** Surgeon participation and adherence to study phases

Surgeon	Pre-Intervention Cases	Post-Intervention Cases	Total Cases	Status
Surgeon 1	5	1	6	Followed both phases
Surgeon 2	6	3	9	Followed both phases
Surgeon 3	4	2	6	Followed both phases
Surgeon 4	6	4	10	Followed both phases
Surgeon 5	2	1	3	Followed both phases
Surgeon 6	1	1	2	Followed both phases

### Overall positive but highly variable utilization change

The overall trend showed a positive outcome, with an average utilization increase of over 63% across the cohort (Table 2). This indicates that the intervention, despite its flaws, was associated with a desirable reduction in instrument waste.

**Table 2:** Overall mean instrument utilization change

Entity	Pre-Utilization	Post-Utilization	Delta	Percentage Change
All Procedures (Mean)	0.335	0.548	+ 0.213	+ 63.6%

However, this positive result masks extreme variability, which forms the core of the unreliability argument. The Delta in utilization varied by over 300% across the surgeons and showed significant inconsistency across procedures.

### Variability by surgeon

The increase in utilization varied dramatically across the six surgeons (Table 3), ranging from a modest increase of Delta = + 0.103 to a massive increase of Delta = + 0.418.

**Table 3:** Variability in utilization change by surgeon (sorted by delta)

Surgeon	Pre-Utilization	Post-Utilization	Delta	Percentage Change
Surgeon 1	0.437	0.540	+ 0.103	+ 23.6%
Surgeon 2	0.366	0.525	+ 0.160	+ 43.6%
Surgeon 3	0.362	0.533	+ 0.171	+ 47.2%
Surgeon 4	0.336	0.577	+ 0.241	+ 71.6%
Surgeon 5	0.158	0.548	+ 0.390	+ 246.8%
Surgeon 6	0.168	0.586	+ 0.418	+ 248.1%

The difference between the lowest and highest Delta suggests that the intervention’s success is highly dependent on the individual surgeon, making the overall approach unpredictable and therefore unreliable for a system-wide solution.

### Variability by procedure

The effect was also inconsistent across procedure types (Table 4). While most procedures saw a positive Delta, one procedure (GT) showed a slight decrease in utilization (Delta = -0.052). This negative Delta suggests an increase in waste for that specific procedure, indicating that the human-based intervention failed to produce a positive outcome universally.

**Table 4:** Variability in utilization change by procedure (sorted by delta)

Procedure	Pre-Utilization	Post-Utilization	Delta	Percentage Change
GT	0.610	0.558	-0.052	-8.5%
IHR	0.607	0.667	+ 0.060	+ 9.8%
Appe	0.184	0.521	+ 0.337	+ 182.9%
UHR	0.172	0.560	+ 0.388	+ 225.8%

### Statistical comparison of pre- and post-intervention utilization

Paired nonparametric analysis using the Wilcoxon signed-rank test confirmed that the observed variability across surgeons and procedures was statistically meaningful. When aggregated by surgeon, instrument utilization showed a significant median decrease of 0.25 (95% CI − 0.40 to − 0.13, *p* = 0.028), corresponding to a large rank-biserial effect size (*r* = 1.0). At the procedure level, the median change (–0.19) did not reach statistical significance (*p* = 0.144) because of limited sample size (*n* = 4) despite a similarly large effect (*r* = 0.9). When both surgeon and procedure were jointly considered, the median reduction of 0.27 (95% CI − 0.36 to − 0.09, *p* = 0.0076, *r* = 0.96) was associated with a post-intervention decrease in utilization. These findings indicate that although the direction of change was uniform across most pairings, the magnitude and reliability of the human-based intervention remained inconsistent, reinforcing the limitations of manual compliance and self-reported data capture.

## Discussion

### Reliability of human-based interventions

The results suggest that while a directional increase in instrument utilization was observed among documented cases, the human-based intervention and its associated data collection were fundamentally unreliable for sustainable and predictable waste reduction. The unreliability stems from two critical factors: inconsistent behavioral change and flawed data fidelity.

First, the wide range in Delta across surgeons, from + 0.103 to + 0.418, together with a negative Delta for one procedure, illustrates the inconsistency of human behavioral response to the intervention. The observed improvements were strongly surgeon-dependent, indicating that education, feedback, or external pressure did not translate into a uniform or sustained change in practice across participants. In the operating room, where patient safety appropriately takes precedence, clinicians may continue to open full trays, thereby limiting the effectiveness of waste-reduction initiatives [11]. At the same time, a critical competing explanation for the largest observed utilization increases, particularly those exceeding 200%, is a change in documentation accuracy rather than true behavioral modification. In human-reported systems, under-documentation of used instruments in the pre-intervention phase may artificially lower baseline utilization, while subsequent improvements in reporting completeness alone could produce large apparent gains without corresponding changes in intraoperative practice. Importantly, whether the observed deltas reflect behavioral change or improved documentation accuracy, both interpretations support the same conclusion that human-dependent data collection introduces substantial uncertainty and undermines reliability.

Second, and most critically, the study’s reliance on human-reported utilization data introduces significant issues regarding data fidelity, consistency. This is illustrated by the data capture rates, falling from an already low 24.7% to a mere 15.3% post-intervention. This indicates a systemic failure in data collection by staff, possibly stemming from a lack of commitment from overworked and understaffed OR personnel who see no direct benefit from participating in such studies. Such a phenomenon is not unique; it suggests a pervasive bias in many human-led instrument reduction studies, where the data collected may represent only the most engaged participants rather than the general population.

The user’s concern about non-adherence is statistically supported by the imbalanced case volumes (Table 1) and the concentration of data from just two surgeons. Furthermore, the extreme positive Delta values for Surgeon 5 and Surgeon 6 (over 240% increase) suggest a possible shift in the method of data collection or reporting rather than a genuine, massive change in utilization behavior. In either case, whether the intervention failed to achieve consistent behavioral change or failed to secure accurate data, the human-based approach of data collection, particularly that relies of staff, appears to be unreliable [12]. This aligns with findings in other surgical human reliability studies that document the high error rates associated with human observation in the OR [13].

### Is now the time for computer vision integration?

The limitations observed in this human-based optimization initiative highlight the need for more objective and reliable approaches to instrument utilization measurement. Inconsistent participation, declining data capture, and wide variability in outcomes suggest that reliance on manual documentation and behavioral compliance may constrain the scalability and reproducibility of tray optimization efforts across institutions.

In this context, computer vision (CV) technologies represent a promising avenue for improving data fidelity in the operating room. CV systems that leverage cameras and deep learning models have demonstrated the ability to detect, track, and count surgical instruments in real time across a range of experimental and clinical environments [9, 14].

CV-based approaches offer several potential advantages:

- Objective Data Fidelity: CV may reduce reliance on manual counting or self-reporting by providing continuous, verifiable records of instrument usage, which could improve accuracy in tray optimization and intervention evaluation [10].
- Consistency and Adherence: Automated systems operate independently of human compliance, enabling more consistent data capture across surgeons, procedures, and cases, thereby potentially reducing variability observed in manual reporting.
- Real-Time Optimization: CV platforms may support immediate feedback to surgical and sterile processing teams, allowing process adjustments that extend beyond retrospective human documentation [15].

Importantly, while CV approaches have shown feasibility in prior studies [16, 17], their integration into routine perioperative workflows remains an area of ongoing research. The present findings do not evaluate CV directly but instead underscore the limitations of human-centered data collection strategies that CV-based systems are designed to overcome. Future studies comparing manual and automated approaches prospectively will be essential to determine the extent to which CV can improve reliability, reduce waste, and support sustainable tray optimization.

### Limitations

This study should be interpreted in light of several important limitations. First, the pre–post observational design lacked randomization, blinding, and a parallel control group, which limits causal inference regarding the effectiveness of the tray optimization initiative. As is typical in quality improvement interventions within operative settings, the study relied on before–after comparisons that are inherently vulnerable to temporal confounding and selective participation.

Second, data capture was incomplete and declined over the course of the study, reflecting inconsistent adherence to manual reporting by operating room personnel. This low fidelity limits the representativeness of the analyzed cases and introduces potential reporting bias, whereby highly engaged individuals may have been more likely to contribute data. Consequently, observed utilization changes may partially reflect variation in documentation practices rather than true behavioral change. These limitations primarily reflect failures of human-dependent informatics and compliance systems rather than deficiencies in surgical clinical judgment; surgeons may appropriately prioritize patient safety by opening full trays, while unreliability arises from inconsistent manual documentation and reporting burden placed on perioperative staff.

Third, the analysis was restricted to standardized instrument sets and excluded individually wrapped instruments and disposable items. While this focus aligns with the scope of the optimization initiative, it underestimates total instrument waste and limits assessment of broader perioperative resource utilization.

Fourth, the study involved a small number of surgeons at a single institution and focused on selected high-volume procedures with relatively consistent instrumentation requirements. Although this approach facilitated tray standardization, it limits generalizability to other surgical specialties, institutions, and procedures with higher case-to-case variability.

Finally, variability in participation across surgeons and procedures resulted in imbalanced sample sizes, reducing statistical power and limiting the ability to detect consistent effects across subgroups.

Despite these limitations, the study provides important insights into the practical challenges of human-based tray optimization efforts, particularly regarding data fidelity, adherence, and outcome variability, which are central to the interpretation of reported improvements in utilization.

## Conclusions

Despite a directional increase in instrument utilization was observed among documented cases following a human-based tray optimization initiative, critically low and declining data capture, substantial participation imbalance, and extreme outcome variability limit interpretability. Large observed utilization changes may reflect shifts in documentation accuracy rather than consistent behavioral change, underscoring the inherent unreliability of human-dependent data collection in the operating room. These findings indicate that scalable and reproducible waste reduction efforts will likely require objective, automated measurement approaches such as computer vision rather than continued reliance on manual compliance and self-reported utilization.

## Data Availability

Data and materials will be provided by the authors upon reasonable request.
